# Supplementary material for: A Computational Model for Estimating the Progression of COVID-19 Cases in the US West and East Coast Population Regions
Source: Exp Results. 2020 Aug 20;1:e41. doi: 10.1017/exp.2020.45 (PMC7557233; doi:10.1017/exp.2020.45)
Supplement: Supplementary file 1 [file S2516712X20000453sup002.pdf › S2516712X20000453sup002.pdf]

The Editorial Board
Experimental Results

Dear Dr. Henrion,

We wish to submit the revised manuscript for "A Computational Model for Estimating the Progression of COVID-19 Cases in the US West and East Coasts" to be considered for publication in Experimental Results. We are thankful for the responses by the reviewers, and here we would like to address the comments made point-by-point (our responses in blue and detailed changes to the manuscript in red).

<u>Reviewer #1:</u>

The authors used SEIR model to describe COVID-19 outbreak in the US. Their approach relied on numerous assumptions most of which are not justified/supported by empirical data; the validity of their simulation results is questionable. The 25% infected scenario analysis seems to be merely a circular argument; the authors assume that the 25% of the population will be infected and then simulate the epidemic to show that almost 25% of the US population would be infected. The results for the test-and-isolate scenario relied on a very optimistic assumption that the hospitalized individuals are fully isolated regardless of the medical capacity overwhelmed by the surge in the test positive individuals, which would never be true in reality. The results that the authors present in the manuscript would not be of actual epidemiological interest. Given the overall lack of technical and contextual soundness, I regret to note that I did not find added value in this study to the scientific literature.

We thank Reviewer #1 for the comments on our manuscript. We agree that we made some assumptions during the study, but it is important to note that our study was completed during the very initial stages of COVID-19 surge in the United States (early March). At that point, there were no models projecting COVID-19 in the US, and the empirical data that Reviewer #1 requested was unavailable. Thus, we had to rely on data obtained from other countries to serve as estimates, and the data were already relatively low (e.g. Lazzerini et al., 2020). We are aware that our model has limitations which we mentioned in Lines 142 – 144: *"Our model and projections can be further improved when transmission dynamics in the US begin to be documented and governmental response begin to be properly executed accordingly*". While it is unfortunate that the reviewer did not find added value in this study to the scientific literature, we know that some aspects of the manuscript have been of interest to other researchers.

We agree that the test-and-isolate assumption the reviewer raised may have appeared quite optimistic. A surge in test-positive individuals would certainly overwhelm medical capacities. However, the optimistic assumption is actually quite feasible if responses were strict enough, as seen from the way other countries have handled COVID-19 (such as how South Korea suppressed its surge and Singapore's health system), or even during the sporadic outbreaks of contagious diseases in the US. Also, to reliably estimate healthcare capacity, it requires data which are not easily accessible. Nevertheless, we agree with the reviewer and a much more accurate model can be obtained if it was possible to project healthcare capacities accurately, especially if cases each exceptionally high. This is a very feasible future work from this manuscript. Thus, we have added the following statement into the discussion section of our manuscript as follows:

In addition, our test-and-isolate scenario is also quite optimistic as we assumed that hospitalized individuals can be fully isolated regardless of medical capacity despite a potential surge in COVID-19 patients. While some countries have been able to successfully contain COVID-19 by relying on efficient tests and isolation, it remains uncertain whether the US is able to similarly do so in light of constrains to testing, healthcare supplies, and capacities (Yeo and Ganem, 2020).

We are also aware and understand that Reviewer #1 might have overlooked the time this study was conducted due to the relatively late timing of our submission. The reasons have been explained in the *Cover Letter* during our original submission. Nevertheless, we thank the reviewer for the comments which provided us an opportunity to justify our work accordingly.

Minor comments:
- There may have been flaws in their model or coding: for example, the epidemic curve is growing rapidly even in Phase 3, when R = 1 and thus theoretically the epidemic should not be growing.

We respectfully disagree with the reviewer's comments. R represents contagiousness of a disease and does not indicate that the *cumulative amount* of infected people should start to decrease immediately when $R = 1$. It depends on the set-up of the ODEs (refer to Methods). In our case, as long as $\alpha\rho E > \gamma I_H$ and $\alpha(1-\rho)E > \gamma I_C$, the curves will continue to rise. We will require a few iterations after the onset of setting $R = 1$ for $\frac{dE}{dt} < 0$ and $|\frac{dE}{dt}|$ large enough so that E reduces sufficiently before the curves of I start to decrease, i.e. $\alpha\rho E < \gamma I_H$ and $\alpha(1-\rho)E < \gamma I_C$.

- The 25% scenario: how could the authors justify the final size of COVID-19 and pH1N1 would be the same, when R0 of the former is almost twice as that of the latter?

The 25% infected for pH1N1 was based on a meta-analysis of serological studies (Kerkhove et al., 2013) and not epidemiological studies; likewise, R_0 only gauges contagiousness and does not represent the final size of people infected. We can agree that the 25% scenario section may seem to appear out of nowhere and was too broad of a prediction especially during the very initial stages of COVID-19, but we included it to show how our model could be applied to a broader context. We will consider removing it if other reviews find this section redundant.

Reviewer #2:
Interesting work. I suggest minor revisions in 3 specific areas:
1) precision of wording (eg. east and west coast "population regions"), also could you clarify what cities you have included in these areas

We agree with the comments on the wording and edited the manuscript accordingly: "… West and East Coast population regions" have been included for the Title, Abstract, and Discussion. We did not edit the remaining sections to maintain conciseness.

Regarding the cities, there are unfortunately too many cities to include. The cities we considered are within the state boundaries defined in the US Census Bureau, and we checked the geographic location of every reported case to ensure they fall within the boundaries of interest.

2) layout of the paper, put equations in order with each assigned a number for reference, I suggest you find a good example in Journal of Theoretical Biology and use it as a template,

The reviewer makes a very a good suggestion here. However, we did not need to reference any specific equations throughout the manuscript and all the equations are inter-related and do not represent much alone. As a result, we found no need to assign a number for each equation, as they should be considered as a set.

3) result figure (graphs) could be split apart and/or produced in colour to aid understanding. Its not clear to me what the multiple lines per graph are, or why they are there? Inline with "reproducible research", can you put your code in Annex?

The multiple lines per graph represent each of our 50 simulations. We understand the reviewer's concern, and thus to clarify, we included the following sentence into each figure caption: Each line represents one simulation. We have also included our code in Supplementary Materials.

Minor wording changes in abstract, "make" worst case scenario, change to "estimate" worst, computation to simulation, new cases per day? In the introduction you switch from past to present tense, change all to one or the other.

We have made changes to the Abstract and the Introduction according to the Reviewer's suggestions. For the Introduction, we used different tenses to maintain consistency with the timeline of events.

In the methods, state as many of the explicit assumptions as you can. Why did you choose an Erlang distribution over other more common choices? Reference for 8-10 delay in reporting? Results; exchange word "cool down" for reduction. Pg 6 ln 147 change "simulations" for predictions.

We chose an Erlang distribution for our stage durations because it reflects the distribution of independent and identical normal distributions, and thus, allows us to express our modified SEIR compartmental model neatly in terms of ODEs. Other arbitrary distribution will require integro-differential equations.

Regarding our selection for 8 to 10 days delay in reporting, it was ultimately chosen for these reasons:

i) In the cited study (Lin et al., 2020), they had an estimated 14-day delay because it matches their simulation results. For our case, a range of 8 to 10 days matched our simulation results.

ii) Information on delays in reporting appears to be elusive for COVID-19, and reporting delays are usually too variable for several reasons (e.g. nature of the disease and healthcare systems).

iii) Delay distribution was found to have a mean of 9 days for respiratory viral diseases (measles and mumps) which remarkably aligns within our overall simulations elsewhere (Marinović et al., 2015). Although the study was conducted in the Netherlands and the viruses were distinct from coronavirus, (a) reliable information on reporting delays for COVID-19 are scare; (b) measles and mumps viruses, much like SARS-CoV-2, all have certain unique distinctive characteristics

As a result, we agree with the Reviewer that there is a need to justify our selection of 8 to 10 days reporting delay. We have included the following statement in the Discussion section:
We arrived at an 8 to 10 days reporting delay in a method similar to (Lin et al., 2020). While this is an important factor to consider in disease modeling, the actual reporting delay of COVID-19 is currently too early to tell, and reporting delays usually show very high variance for many reasons (e.g. nature of the disease, transparency of healthcare systems). It may be worthwhile to note that some common distinctive respiratory viral diseases were found to have a mean of 9 days in reporting delay elsewhere (Marinović et al., 2015), a value which aligns with our simulation estimates.

We agree with the other suggestions made by the Reviewer and edited our manuscript accordingly.

Finally you have done good work and can legitimately make a stronger case/statement for increased levels of covid testing and surveillance. Good work, well done.

We wish to thank the reviewer for the valuable and constructive feedback.
